# Structural modification of P-glycoprotein induced by OH radicals: Insights from atomistic simulations

**DOI:** 10.1038/srep19466

**Published:** 2016-02-09

**Authors:** N. Khosravian, B. Kamaraj, E. C. Neyts, A. Bogaerts

**Affiliations:** 1 Research Group PLASMANT, Department of Chemistry, University of Antwerp, Universiteitsplein 1, B-2610 Antwerp, Belgium

## Abstract

This study reports on the possible effects of OH radical impact on the transmembrane domain 6 of P-glycoprotein, TM6, which plays a crucial role in drug binding in human cells. For the first time, we employ molecular dynamics (MD) simulations based on the self-consistent charge density functional tight binding (SCC-DFTB) method to elucidate the potential sites of fragmentation and mutation in this domain upon impact of OH radicals, and to obtain fundamental information about the underlying reaction mechanisms. Furthermore, we apply non-reactive MD simulations to investigate the long-term effect of this mutation, with possible implications for drug binding. Our simulations indicate that the interaction of OH radicals with TM6 might lead to the breaking of C-C and C-N peptide bonds, which eventually cause fragmentation of TM6. Moreover, according to our simulations, the OH radicals can yield mutation in the aromatic ring of phenylalanine in TM6, which in turn affects its structure. As TM6 plays an important role in the binding of a range of cytotoxic drugs with P-glycoprotein, any changes in its structure are likely to affect the response of the tumor cell in chemotherapy. This is crucial for cancer therapies based on reactive oxygen species, such as plasma treatment.

P-glycoprotein is an important protein present in the plasma membrane of human cells. It is strongly expressed in tumor cells and causes reduced access of cytotoxic drugs in these cells^1^. It is believed that the treatment failure in over 90% of the patients with metastatic cancer is mainly due to a failure in response to chemotherapy by acquiring multidrug resistance due to the function of P-glycoprotein^2^. Indeed, P-glycoprotein acts as an efflux transporter pump responsible for the resistance against drug delivery^3^.

This protein is composed of 1280 amino acids and is expressed as a 170–180 kDa plasma membrane. It has two symmetrical nucleotide binding domains (NBDs) and transmembrane domains (TMDs), including six α-helical membrane-spanning domains^4^,^5^,^6^. The active pore, through which drugs are exported, is formed by twelve TM segments and NBDs oriented in the cytoplasm. Among the twelve TM domains in P-glycoprotein, transmembrane 6 (TM6) plays a critical role in binding substrates such as cytotoxic drugs (e.g. vinblastine and actinomycin D) to P-glycoprotein^7^,^8^. This membrane protein is highly exposed to reactive oxygen species (ROS) originating from an external source, as e.g. induced by radiation or from non-thermal atmospheric plasma, which is also an effective source of ROS in cancer treatment^9^.

In this paper, we computationally investigate the possible interaction of OH radicals with TM6, in order to understand its possible effect on the structure of this protein domain, with implications for its drug binding affinity. We have chosen to focus our study on OH radicals since these are the most effective ROS in biological systems^10^,^11^. The envisaged understanding is crucial for the combination of cancer therapies based on ROS, such as plasma treatment, with chemotherapy, as this combination might reduce the tumor cell resistance against chemotherapy.

The interaction of ROS with proteins leads to protein and peptide oxidation, degradation, mutation, fragmentation and destruction, which all affect the overall function of the protein^12^,^13^,^14^. Several experimental approaches have been implemented in literature to study the most reactive sites of proteins, peptide and amino acids upon impact of ROS^15^,^16^,^17^. Although experiments are indispensable in this field, they are not able to fully describe the reaction dynamics and pathways contributing to fragmentation and mutation at the molecular level.

Computational studies, especially based on atomistic simulations, have significantly contributed to our understanding in the underlying interaction mechanisms of free radicals with peptides. Quantum mechanical (QM) calculations have been performed to study the interaction of OH radicals with alanine (Ala) and glycine (Gly)^18^, L-phenylalanine (L-Phe)^19^, as well as the interaction of peroxy radicals with a peptide composed of Ala and Gly^20^. QM calculations have also been used to study H-abstraction from an α-carbon site in leucine (Leu) dipeptide^21^. Such calculations, however, are computationally very demanding, and are hence invariably limited to small peptides.

To overcome this limitation, approximative QM methods, such as the so-called self-consistent charge density functional tight binding (SCC-DFTB) method can be applied. Results obtained using SCC-DFTB simulations are generally in very good agreement with the results from B3LYP-based DFT calculations^22^,^23^. DFTB was also applied in quantum mechanical/molecular mechanics (QM/MM) approaches, e.g., for energy optimization and trajectories of biomolecules in solution^24^,^25^,^26^,^27^. So far, however, there are no studies yet reporting on the reactivity of actual peptides with a size comparable to TM6 at the QM level.

In this paper, we apply for the first time the SCC-DFTB method to investigate the interaction of OH radicals with the TM6 domain of P-glycoprotein. The aim is to find the most reactive sites of TM6 that induce fragmentation and mutation upon impact of OH radicals, and to elucidate the underlying mechanisms. Moreover, to study the effect of this mutation on the structural behaviour of TM6 with implications for drug binding, we also use non-reactive molecular dynamics (MD) simulations, which are computationally less demanding, and thus able to handle longer time-scales, needed for this purpose.

## Results and Discussion

**Figure 1:**
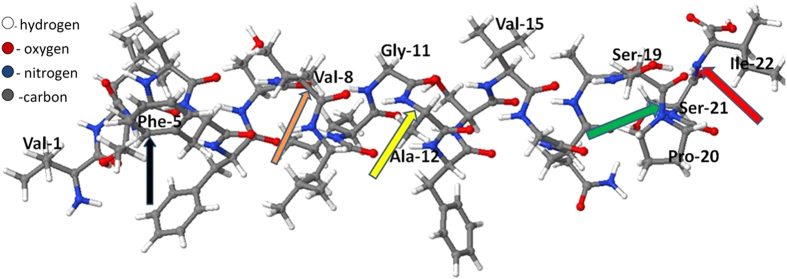
Structure of TM6, consisting of 22 amino acids, represented in ball and stick form. Some of the key amino acids are labelled. The yellow arrow line points towards the breaking of a C-N bond between Ala-12 and Gly-11 (cf. Figure S1), the red arrow line points towards the breaking of a C-N bond between Ser-21 and Ile-22 (cf. Fig. 2), the green arrow line points towards the breaking of a C-N bond between Pro-20 and Ser-19 (cf. Fig. 3), the brown arrow line points towards the breaking of a C-C in Val-8 (cf. Figure S2). Finally, the black arrow line points towards the mutation of Phe-5 at site p.

We first investigate all possible reactions in the backbone and side chains of TM6, leading typically to oxidation, fragmentation or detachment of small molecules. Subsequently, we explain the mechanism of mutation in the aromatic ring, as well as its long term effect on the structure of TM6. The TM6 structure of P-glycoprotein is taken from the Uniprot database (Uniprot ID:P08183) and is illustrated in Fig. 1. It contains 22 amino acids, of which some important ones are indicated in Fig. 1.

Based on in total 50 simulation runs (each containing 10 OH radicals), we find the probability of interaction with the backbone to be 1.3 times higher than for interaction on the side chains. The relevant statistical results are given in Table S1 of the Supporting Information.

### Reactions in the α-site of the backbone

Our simulations indicate that OH radicals are able to abstract an α-hydrogen atom of all amino acids except Gln within the TM6 structure. The reason that no H-abstraction was observed for Gln is probably simply due to the limited statistics. However, it can also be due to the existence of a higher reactive side chain (NH_2_) group in Gln. Our simulations indeed seem to indicate that the reactions in the side chain of Gln occur more frequently; thus the interaction of OH radicals with NH_2_ are more favoured than with the α-site of Gln.

The consequence of this H-abstraction is the creation of an α-carbon radical that initiates peptide oxidation. Indeed, although the latter can be stabilized by delocalizing the unpaired electron to the neighboring functional groups, it can also easily react with other free OH radicals in the system, yielding a hydroxylated α-carbon^28^. Subsequently, our results indicates that the latter can react with other free OH radicals, again resulting in H-abstraction and formation of a carbonyl group on TM6, yielding backbone oxidation and subsequently peptide bond cleavage of TM6, in agreement with one of the feasible reaction pathways for OH/O_2_ impacts on peptide^10^.

Besides the fragmentation as a result of oxidation, our results also show that the α-carbon radical can form a double bond with its adjacent nitrogen atom, prone to peptide bond cleavage at the other side. This was mostly observed for the peptide bond between Gly (at position 11 of TM6) and its two neighbour residues (i.e., Ile and Ala), and between Gly (at position 16 of TM6) and Val. Gly is reported as a preferential reaction site in peptides upon impact of OH radicals^16^. The mechanism of this fragmentation as obtained from our simulations is shown in Figure S1 of the Supporting Information. In short, the carbon radical has a tendency to pair up again with another electron, resulting in the formation of a double C_3_ = N_4_ bond. As a consequence, the N_4_-C_5_ bond breaks, and thus, fragmentation between Gly and Ala occurs. The same mechanism is also observed for the impact of OH radicals on Phe and Ser as potential fragmentation sites in TM6.

### Reactions in the side chains and N-site

**Figure 2:**
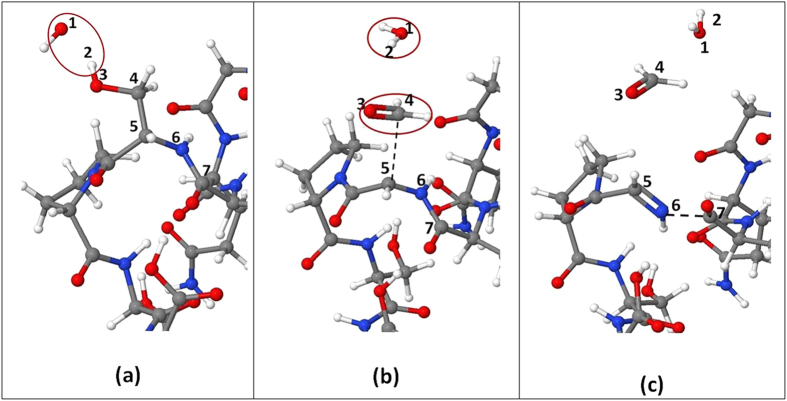
Snapshots from MD simulations, presenting the breaking of a C−N bond in the backbone of TM6 upon impact of an OH radical on the hydroxyl group of Ser. (**a**) The OH radical (red circle) approaches H_2_. (**b**) The OH radical abstracts the H_2_ atom connected to O_3_, forming a water molecule. Subsequently, a double O_3_-C_4_ bond is created, which leads to the dissociation of the C_4_-C_5_ bond and the detachment of formaldehyde (red circle). (**c**) Subsequently, a double C_5_-N_6_ bond is formed and the N_6_-C_7_ bond is dissociated (black dashed line).

The most important reactions of the OH radicals with several functional groups in the side chains and N-site of the various amino acids within TM6 are listed in Table S2 of the Supporting Information. The event with the highest occurrence probability per accessible site is H-abstraction from the OH group of Ser, Thr and Ile at position 22 leading to C-N bond breaking in the backbone. The observed mechanism for the reaction at the hydroxyl site of Ser is explained in Fig. 2. This results in fragmentation between Ser and its adjacent amino acids (i.e., Pro and Ile). In the case of the interaction of OH radicals with the hydroxyl group of Thr at position 3 of TM6, the C-C bond in the side chain is broken and an acetaldehyde molecule is formed.

As presented in Table S2, the second favoured reaction event per accessible site is initiated by the interaction of OH radicals with the H atom at N-site (NH group) of several amino acids, forming a dangling bond on the nitrogen. However, this dangling bond remains stable and no fragmentation of the peptide occurs as a result of this reaction.

The OH radicals can also react with the methyl group of various amino acids (see Table S2), but only the reaction in Ala leads to transfer damage from the side chain to the backbone and eventually fragmentation (i.e., C-C bond breaking). The reaction in Val, Leu and Ile leads to the detachment of various types of hydrocarbon fragments from TM6 as a result of H-abstraction from the methyl group, but not to fragmentation. This reaction mechanism is illustrated for Val at position 8 in Figure S2.

The fourth feasible reaction (as shown in Table S2) is initiated by H-abstraction at β-site of the Ser, Val, Gln, Ile, Phe, Leu, Thr residues. The initial product of this interaction is the formation of a β carbon-radical on the above-mentioned residues, but this radical is stabilized and no damage to the backbone is transferred.

**Figure 3:**
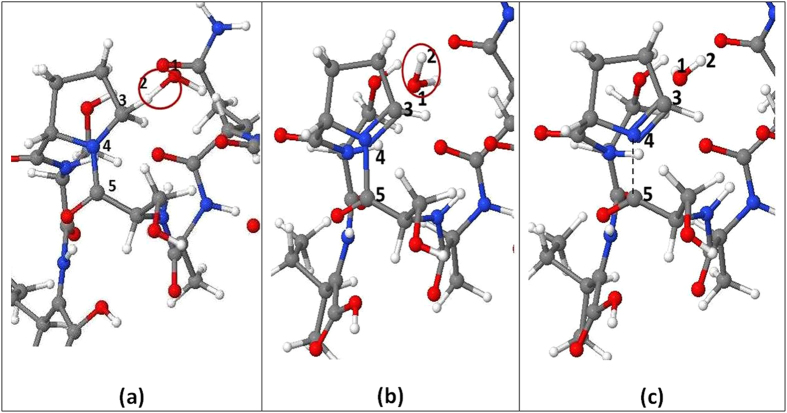
Snapshots from MD simulations, presenting the breaking of a C−N bond in the backbone of TM6 upon impact of an OH radical on the H atom in the ring of Pro. (**a**) The OH radical (red circle) binds to H_2_. (**b**) The OH radical abstracts the H_2_ atom connected to C_3_, forming a water molecule (red circle). (**c**) Subsequently, a double C_3_-N_4_ bond is created, which leads to dissociation of the N_4_-C_5_ bond (black dashed line).

The third reaction that causes peptide bond cleavage, more specifically between Pro and its neighbour amino acid (Ser), is initiated by the interaction of OH radicals with hydrogen in the ring of Pro. The reaction mechanism is explained in Fig. 3.

It is clear that all reactions are initiated by H-abstraction, forming a water molecule. Depending on the position, and the stabilization of the resulting radical, this reaction can either lead to peptide bond cleavage or not. The last two types of reactions indicated in Table S2 are with Phe. They do not give rise to peptide bond cleavage, but they can yield mutation, as will be described in section 3.4 below.

### Statistics on peptide fragmentation in TM6

We analyzed the simulation results of in total 50 runs, corresponding to 500 OH radical impacts, by investigating the changes in C-C and C-N bonds in the backbone of TM6 upon impact of OH radicals (see Table S3). In total, 97 of the observed reactions (i.e., almost one fifth of all reactions) lead to C-C or C-N bond breaking in the backbone. Furthermore, the OH radicals are slightly more effective in breaking C-N bonds than C-C bonds.

In general, peptide fragmentation is an important consequence of ROS impacts, and strongly depends on the type and arrangement of the amino acids in the peptide, which determines the sites in the peptide where damage occurs^10^. Our results suggest that TM6 contains several reactive sites, which might be affected by the impact of OH radicals and subsequently they may induce fragmentation in TM6. Due to the main role of TM6 in drug binding, fragmentation of this domain might significantly disturb the drug resistance as a main function of P-glycoprotein.

### Mutation in TM6

**Figure 4:**
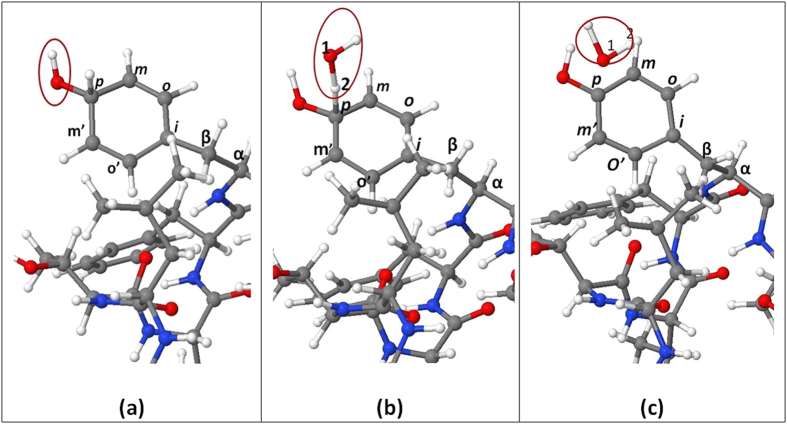
Snapshots from MD simulations, presenting the impact of an OH radical on the aromatic ring of Phe in TM6. (**a**) The OH radical attaches to the para site of the aromatic ring. (**b**) Another OH radical approaches and binds to the H_2_ atom of the para site (red circle). (**c**) H_2_ is abstracted by the OH radical, forming water (red circle) and converting Phe into Tyr.

In addition to fragmentation, the OH radicals may also yield mutation in one of the residues, which can have significant consequences. Our simulations reveal a possible mutation in Phe upon OH impact. Phe is located at positions 5, 6 and 13 in the TM6 structure. This aromatic residue is very important, because of its role in the interaction with drugs. We observed that OH radicals can react with the ipso, ortho, meta and para sites of the aromatic ring, indicated as I, o, m and p in Fig. 4. Both H-abstraction and OH addition are seen to occur at the various sites. This induces changes in the aromaticity, which will probably lead to a change in the binding energy with the drugs. Furthermore, when OH addition and H-abstraction occur at the same position in the aromatic ring, which happened 8 times in our 50 simulations (500 impacts), a new amino acid, i.e., tyrosine (Tyr), is formed in our simulations. This process is shown in Fig. 4. The OH radical forms a bond with C_2_ in the para site of the aromatic ring (red circle in a). Another OH radical abstracts the H_2_ atom on this para site (b), forming again a water molecule (c). As a result, the aromatic ring has changed, and thus, Phe is transformed into Tyr. The same product was also experimentally observed in the hydroxylation of Phe by the Fenton reaction and γ-radiolysis^29^. This mutation might have a crucial effect on the general function of TM6, as will be elaborated in the next section.

### Effect of mutation on the structure of TM6

The above analysis reveals a novel possible mutation (called F335Y) on TM6 of P-glycoprotein. We therefore applied non-reactive MD simulations to elucidate the structural impact upon this mutation on longer time scales. We analyzed the DSSP, helicity fraction, RMSD and RMSF, and we performed PCA analysis, for both the native and mutant (F335Y) structures of TM6.

**Figure 5:**
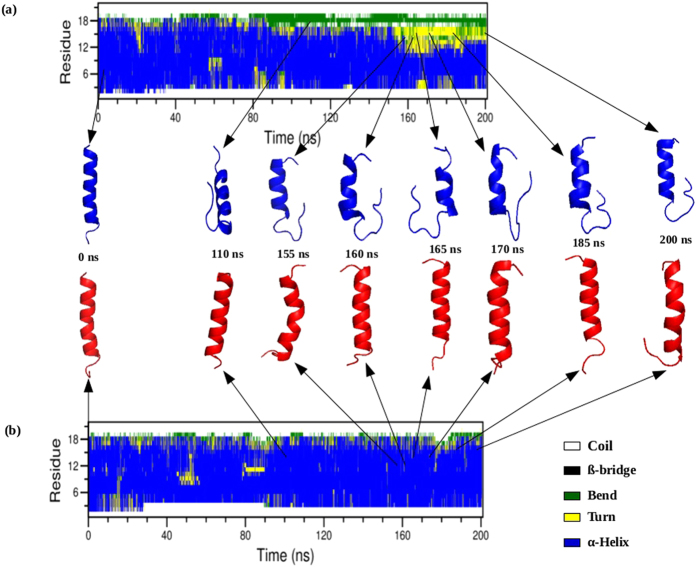
Time evolution of the distribution over secondary structural elements of TM6 of P- glycoprotein at 300K (DSSP classification) and snapshots of native and mutant (F335Y) TM6 of P-glycoprotein conformations, at different times. (**a**) Native and (**b**) mutant (F335Y).

Figure 5 shows the distribution of secondary structural elements (i.e., coil, turns, bend, β-bridges, α-helix and π-helix) in both the native and mutant structures of TM6, as a function of time, as obtained from the DSSP analysis during the entire simulation time. It is clear that the α-helix is the dominant conformation, especially in the mutant structure of TM6. The native structure shows a significant fraction in bend conformation for the residue positions 347–349 (indicated as 17–19 in Fig. 5) after 80 ns till the end of the simulation, as well as in turn conformation for the residue positions 342–346 (indicated as 12–16 in Fig. 5) after 150 ns till the end of the simulation. As the α-helix is typically more stable and rigid in nature than the other secondary structures, we may conclude that upon mutation, TM6 loses its flexibility, and becomes more rigid.

The structures of both the native and mutant TM6 at a time of 0 ns, 110 ns, 155 ns, 160 ns, 165 ns, 170 ns, 185 ns and 200 ns are also indicated in Fig. 5. It is indeed clear that the mutant structure (red) is more rigid (more α-helix) than the native structure (blue).

The latter can also be deduced from Figure S3, which presents the fraction of time during the entire simulation that the native and mutant structures of TM6 are in helical structure. The difference between both is small, especially for residue positions 4–10 and 18–20, but for the residues positions 1–4 and 11–18, the mutant structure has a higher helical fraction than the native one, which confirms that the mutant structure is more rigid in conformation than the native structure of TM6.

The higher rigidity of the mutant TM6 structure is also clear from the RMSD and RMSF plots of the backbone Cα-atoms of both the native and mutant structures, presented in Figures S4 and S5. The RMSD plot (Figure S4) indicates that the native and mutant structures show a similar deviation until ~89 ns, but then the native structure starts deviating more than the mutant structure, indicating that it is more flexible. This is also clear from the RMSF plot (Figure S5), where the native structure again shows a somewhat higher degree of flexibility than the mutant structure, more specifically in residues 335 to 352 (indicated in Figure S5). The time-averaged RMSD and RMSF values of both the native and mutant structures of TM6 are presented in Table 1. The lower values for the mutant structure indeed indicate its higher rigidity.

Finally, we performed a PCA analysis, and the projection of the first two eigenvectors (Figure S6) shows that the mutant structure covers a smaller region of phase space in both the PC1 and PC2 plane than the native structure, again indicating the more rigid structure. The dynamics of the two proteins are best characterized through their phase space behaviour. The eigenvectors of the covariance matrix are called its principal components. The change of a particular trajectory along each eigenvector is obtained by this projection. The overall flexibility of the two proteins is further examined by the trace of the diagonalized covariance matrix of the Cα atomic position fluctuations, and the obtained values for the native and mutant TM6 structures are also listed in Table 1, again confirming the overall lower flexibility in the mutant structure than in the native structure at 300 K. Thus, we can conclude that TM6 loses its flexibility upon mutation, and this will affect its conformation, which may increase the drug binding affinity with P-glycoprotein and possibly lead to a more successful cancer (chemo)therapy. The effect of the above-described mutation (F335Y) has not yet been studied in literature. However, it is well expected that any mutation in the structure of TM6, and in particular on Phe 335, has a significant influence on drug binding, as was previously observed upon substitution of Phe 335 with Ala, leading to a reduction in the protein affinity for vinblastine and actinomycin D^8^.

## Conclusion

This computational study suggests that OH radicals can structurally damage TM6 through reactions in the backbone and the side chains of the various amino acids within TM6. Although OH radicals can interact with the α-hydrogen of Thr, Phr, Ile, Ser, Leu, Pro, Ala, Val and Gly within TM6, this mostly results in the formation of a stable carbon radical, by delocalizing its unpaired electron on the adjacent functional groups. Nevertheless, peptide bond cleavage can be initiated by abstraction of the α-hydrogen of Gly, Ser and Phe, as indicated by our simulations.

Moreover, in our Simulations, OH radicals are observed to react with all chemical groups in the side chains, i.e., the methyl, hydroxyl, β-hydrogen and aromatic groups. Among the possible reactions in the side chains, the interaction of OH radicals with the hydroxyl group of Ser, H-abstraction from Pro, as well as H-abstraction from the methyl group of Ala seem to be the most predominant to initiate peptide bond cleavage. The reactions in the methyl and hydroxyl groups can lead to detachment of various hydrocarbon molecules and small aldehydes.

We therefore conclude that TM6 is prone to damage upon OH impact, which is related to the composition and the exact sequence of the amino acids in TM6. Moreover, OH-induced fragmentation in this domain might induce a change in the main function of P-glycoprotein, i.e., in the efflux capacity of drugs from the cell.

Furthermore, our simulations indicate that the OH radicals can interact with the aromatic ring of Phe, yielding either OH addition or H abstraction. As a result, the aromaticity of the Phe ring will change. Moreover, OH addition and H-abstraction at the same position in the ring can lead to hydroxylation of Phe and thus the creation of Tyr at this position. Since drug binding mainly occurs with the aromatic ring of Phe in TM6, this mutation will probably have an influence on its binding energy with drugs.

To study the long-term effect of this mutation on the structure of TM6, we performed non-reactive MD simulations. We analysed the DSSP, helicity fraction, RMSD and RMSF and we performed PCA analysis. The results indicate that TM6 becomes less flexible upon this mutation. This might affect the drug affinity in P-glycoprotein.

In summary, we can conclude that OH radicals seem to be able to induce changes in the structure of TM6, and thus most probably also in the function of P-glycoprotein in its resistance to chemotherapeutic drugs (see Introduction). Indeed, TM6 plays an essential role in drug binding with P-glycoprotein. The overexpression of P-glycoprotein leads to a multi-drug (vinblastine, verapamil, colchicine, etc…) resistance phenotype (efflux mechanism) in various forms of cancer, which is a major barrier to the successful treatment of cancer^2^. Our prediction of this novel possible mutation (F335Y) upon OH impact may contribute to a solution for preventing the efflux of drugs from the cell, as was also demonstrated already in literature for another mutation on Phe at position 335^8^. Therefore, we believe that plasma treatment (or other therapies based on ROS, like OH radicals) may reduce the resistance of cancer cells against chemotherapy, and thus contribute to a better cancer treatment in combination with classical (chemo)therapies, as has already been demonstrated experimentally^30^. In order to validate our model predictions, wet lab data will be required, and we hope that this paper will inspire other groups to perform such experiments.

## Methodology

Approximative QM MD simulations are carried out at the SCC-DFTB level to study the OH/peptide reaction mechanisms. Prior to the MD simulations, the structure is first minimized using steepest descent and subsequently equilibrated at 300 K using the Nosé-Hoover thermostat. To study the reaction mechanisms, 50 MD simulation runs of 10 ps each are performed, employing a time step of 0.5 fs. In each run, 10 OH radicals are initially randomly positioned at a distance of at least 5 Å around the TM6 structure and from each other. Atomic velocities are randomly chosen corresponding to room temperature.

Non-reactive MD simulations are performed using the GROMACS 4.6.3 package^31^, employing the CHARMM27 all-atom force field^32^. Native and mutant structures (F335Y, resulting from the OH-impact) of TM6 are used as input structures, solvated in SPC water molecules. Prior to the MD simulations, the structures are minimized using steepest descent and subsequently equilibrated at 300 K and 1 atm using the Berendsen thermostat and barostat, respectively. The Particle Mesh Ewald (PME) method^33^ is used to treat the long-range electrostatic interactions. The total simulation time is 200 ns, using a time step of 2 fs. The analysis is based on root mean square deviations (RMSD), define secondary structure of protein (DSSP), helicity and root mean square fluctuations (RMSF). To further support our MD simulation results, the large scale collective motion of the native and mutant structures is studied by means of principal component analysis (PCA)^34^. More details about the methods are given in the Supporting Information.

## Additional Information

**How to cite this article**: Khosravian, N. *et al*. Structural modification of P-glycoprotein induced by OH radicals: Insights from atomistic simulations. *Sci. Rep*. **6**, 19466; doi: 10.1038/srep19466 (2016).

## Supplementary Material

Supplementary Information

## Figures and Tables

**Table 1:** Average values of RMSD, RMSF and covariance value of the native and mutant (F335Y) structures of TM6 of P-glycoprotein.

Parameters	Native	Mutant (F335Y)
RMSD (nm)	0.41 ± 0.16	0.26 ± 0.07
RMSF (nm)	0.34 ± 0.15	0.23 ± 0.10
Covariance value (nm^2^)	64.68	37.08
