# Supplementary material for: Structural modification of P-glycoprotein induced by OH radicals: Insights from atomistic simulations
Source: Sci Rep. 2016 Feb 9;6:19466. doi: 10.1038/srep19466 (PMC4746567; doi:10.1038/srep19466)
Supplement: Supplementary Information [file srep19466-s1.pdf]

Supporting Information

Structural modification of P-glycoprotein induced by OH radicals: Insights from atomistic simulations

N. Khosravian*, B. Kamaraj, E. C. Neyts, A. Bogaerts*

Research Group PLASMANT, Department of Chemistry, University of Antwerp, Universiteitsplein 1, B-2610 Antwerp, Belgium

1.Methodology

1.1SCC-DFTB

SCC-DFTB is based on a second-order expansion of the Kohn-Sham total energy around a reference density, in the linear combination of atomic orbitals (LCAO) tight-binding framework [1]. Our simulation setup is as follows. The TM6 structure is placed in the centre of a simulation box with dimensions 52Å×27Å×27Å. The structure of TM6 is shown in Figure 1 of the main paper. It is composed of valine (Val), Leu, thronine (Thr), Phe, serine (Ser), isolucine (Ile), Gly, Ala, proline (Pro) and glutamine (Gln), in the following sequence: Val-Leu-Thr-Val-Phe-Phe-Ser-Val-Leu-Ile-Gly-Ala-Phe-Ser-Val-Gly-Gln-Ala-Ser-Pro-Ser-Ile (i.e., from position 331 to 352 in the P-glycoprotein molecule), as retrieved from the Uniprot database (Uniprot ID:P08183).
TM6 (region 331 to 352) of P-glycoprotein was modeled by I-TASSER server [2] for 3-dimensional structure prediction. This program works by combining the folds and secondary structure by profile-profile alignment threading techniques for non aligned regions. For the submitted sequences, five 3D models were obtained and the best model was selected based on the lowest energy. The modeled TM6 structure is energetically optimized by applying CHARMM27 all-atom force field available under the GROMACS 4.6.3 package. The quality of model structure was verified using the PROCHECK [3] and PROSA [4] programs.
For simplicity, we use numbers 1 to 22 in the main paper, to indicate the position of each residue. Periodic boundary conditions are applied in all directions. The TM6 structure is first minimized and then equilibrated at room temperature by employing the Nosé-Hoover thermostat. The thermostat coupling parameter is set to 100 fs. The steepest descent method is used for minimization with a maximum force component and SCC-tolerance equal to 10^{-4} and 10^{-5} au, respectively.
To obtain statistically valid results for all reaction events, 50 simulation runs are performed. In each run, 10 OH radicals are initially randomly positioned at a distance of at least 5 Å around the TM6 structure and from each other. This distance is chosen to avoid initial interactions between the impinging particles and the TM6 structure. The velocities are randomly chosen and correspond to room temperature. In all simulations (i.e., during equilibration and during particle impact), a time step of 0.5 fs is used. The total time per simulation is 10 ps.

1.2. Non-reactive MD

Non-reactive MD simulations are performed using the GROMACS 4.6.3 package [5]. Native and mutant structures (F335Y; resulting from the OH-impact; see main paper) of TM6 of P-glycoprotein are used as input structures. The systems are solvated in a cubic box with SPC water molecules at 10 Å. At

physiological pH conditions, we make the system electrically neutral in the simulation box. Initially, the solvent molecules are relaxed, whereas all the solute atoms are harmonically constrained to their original positions with a force constant for 5000 steps. Next, the whole molecular system is subjected to energy minimization for 5000 iterations by the steepest descent algorithm, implementing the CHARMM27 all-atom force field [6]. Subsequently, the minimized systems are equilibrated at 300 K by MD in two steps [7], i.e., initially under NVT conditions for 1000 ps, and followed by NPT conditions, also for 1000 ps. During equilibration, all atomic positions are constrained, except for the water molecules, in order to enable a balance of the solvent molecules around the residues of the protein. Temperature and pressure are regulated using the Berendsen thermostat and barostat, respectively. The Particle Mesh Ewald (PME) method [8] is used to treat the long-range electrostatic interactions. The pressure is maintained at 1 atm with an allowed compressibility range of 10^{-5} atm. The LINCS algorithm is used to constrain the bond lengths involving hydrogen, permitting a time step of 2 fs. Van der Waals and Coulomb interactions are truncated at 1.0 nm. The non-bonded pair list is updated every 10 steps and conformations are stored every 0.5 ps. Finally, the systems are subjected to MD simulations for 200 ns each at 300 K without any constraint. We analyse the root mean square deviations (RMSD), define secondary structure of protein (DSSP), helicity and root mean square fluctuations (RMSF), and we make a comparison between the native and mutant (F335Y) structure to examine the structural and functional behaviour of TM6 of P-glycoprotein. To further support our MD simulation results, the large scale collective motion of the native and mutant structures is studied by means of principal component analysis (PCA) [9]. The dynamics of both proteins are best characterized through their phase space behaviour. The eigenvectors of the covariance matrix are called its principal components. The change of a particular trajectory along each eigenvector is obtained by this projection.

2. Supplementary Tables:

Our simulations reveal that the number of interactions on the α-hydrogen atoms of TM6 is significantly lower than the number of interactions on the side chains (i.e., 17.4 % vs 82.6 %), as shown in table 1S. However, by considering the abundance of α-hydrogen atoms in the backbone compared to the number of sites in the side chains, i.e., there are considerably less sites in the backbone than in the side chains (i.e., 24 vs 151), it is clear that the probability of interaction with the backbone is 1.3 times higher than for interaction on the side chains (i.e., 0.725 % vs 0.547% per impact.

Reaction sites	Percentage	Sites	Probability per impact on each site (%)
Backbone (α-hydrogen)	17.4%	22	0.725
Side chains	82.6%	155	0.547

Table S1. Percentage of reactions in the backbone and the side chains

Table S2 lists the important reactions of the OH radicals with several functional groups in the side chains of the various amino acids within TM6. Some of these reactions lead to fragmentation, but not all of them.

Chemical groups	Amino acids	Number of reactions at each site	Accessible sites	Percentage	Probability per impact per accessible site (%)
**OH group**	**Ser,Thr, Ile**	64	6	16.16 %	2.69
N-site	Phe, Gly, Val, Leu, Thr,Alal, Gln, Ser	105	22	26.51 %	1.205
Methyl	Val, Leu, Ile, Ala	101	57	25.50 %	0.447
β site	Ser, Val, Gln, Ile, Phe, Thr, Leu	70	29	17.67 %	0.60
**Pro Ring**	**Pro**	11	7	2.77 %	0.39
H-abstraction from aromatic ring	Phe	29	15	7.320 %	0.488
OH-addition to aromatic ring	Phe	16	18	4.04 %	0.224

Table S2. Occurrence of reactions of OH radicals with different functional groups in the side chains and N-site of the various amino acids within TM6, including the total number of occurrence, the percentage and the probability per accessible site. The reactions that initiate peptide bond cleavage are indicated in bold.

Bonds	Number of broken bonds after 500 impacts	Total number of bonds in 50 runs	Percentage of broken bonds	Percentage of broken bonds per OH radical (%)
C-C	40	1100	3.63 %	0.0073
C-N	57	1050	5.42 %	0.0108

Table S3. Fraction of important bond dissociations (i.e., C−N and C−C bonds)

3. Supplementary Figures:

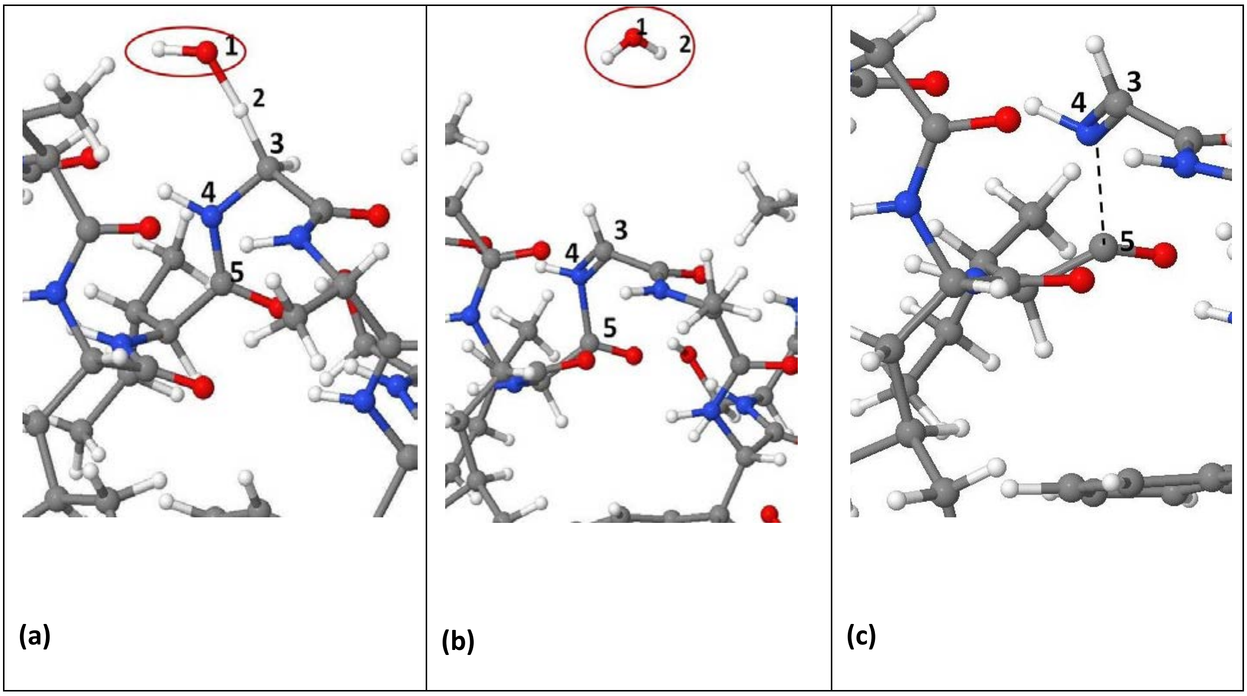

Figure S1. Snapshots from MD simulations, presenting the breaking of a C−N bond in the backbone of TM6 due to OH impact on the α-hydrogen of Gly at position 11. (a) The OH radical (red circle) approaches and binds with H_2. (b) The OH radical abstracts the H_2 atom, which is connected to C_3, forming a water molecule (red circle). (c) A double C_3=N_4 bond is created, which leads to dissociation of the N_4-C_5 bond (see black dashed line).

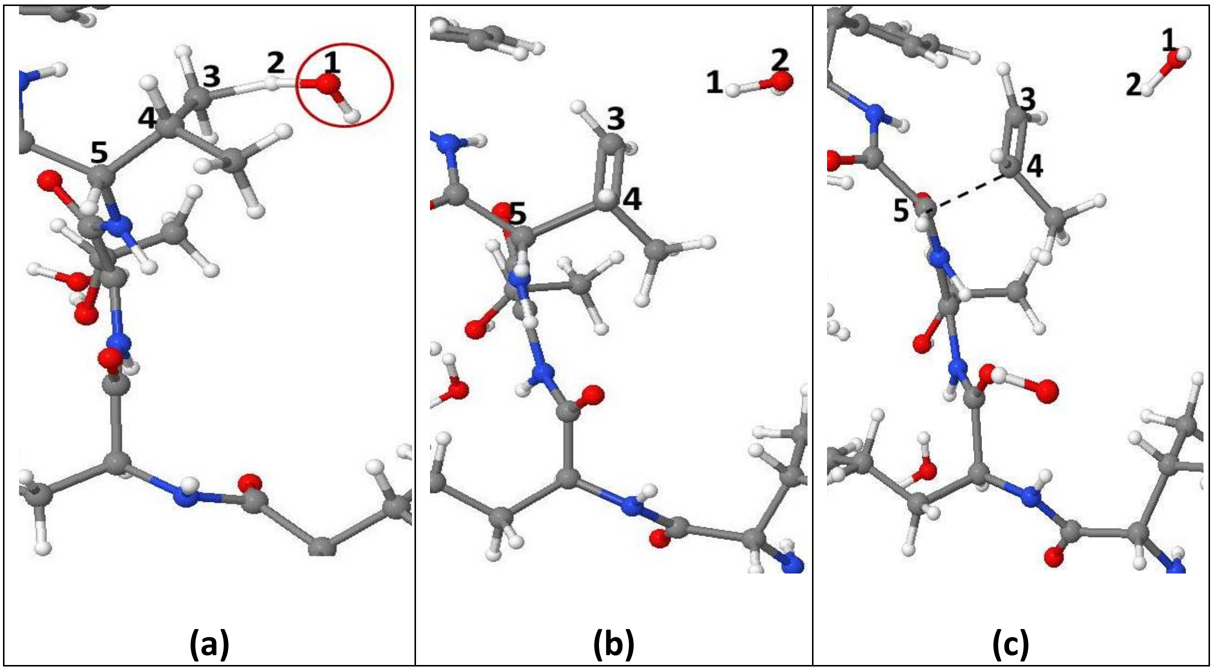

Figure S2. Snapshots from MD simulations, presenting the breaking of a C−C bond in the side chain of Val in TM6 upon impact of an OH radical on the H atom of the methyl site of Val. (a) The OH radical (red circle) approaches, and binds to H_2. (b) The OH radical abstracts the H_2 atomconnected to C_3, forming a water molecule (red circle). (c) Subsequently, a double C_3-C_4 bonds is created, which leads to dissociation of the C_4-C_5 bond and the detachment of propene (C_3H_6) (red circle).

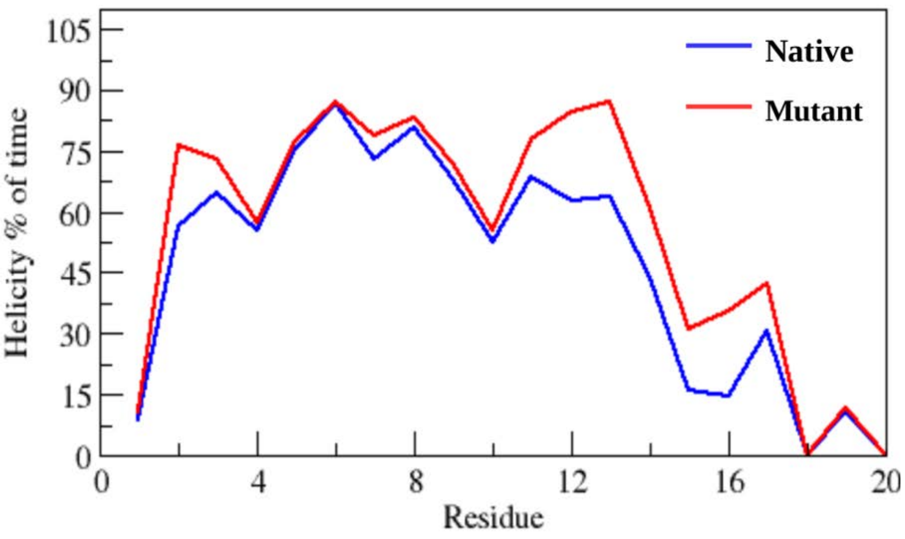

Figure S3.Helicity fraction of time of native and mutant (F335Y) TM6 of P-glycoprotein, for the various residues.

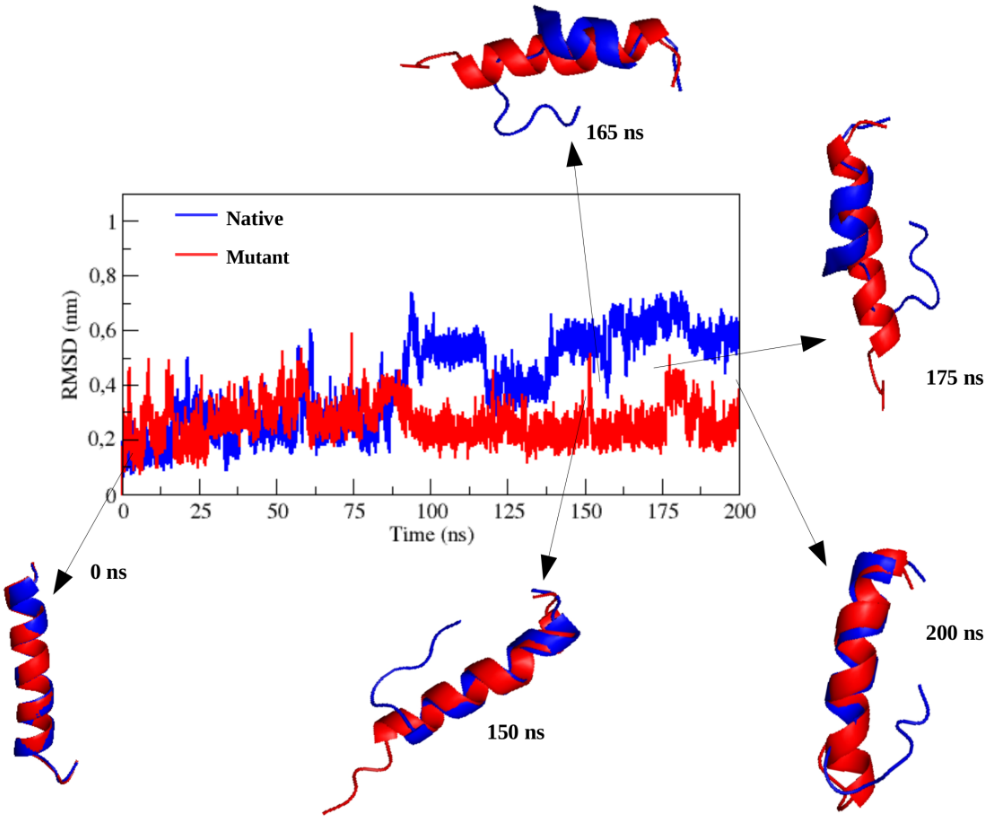

Figure S4.RMSD of native and mutant (F335Y) TM6 of P-glycoprotein versus time at 300K.

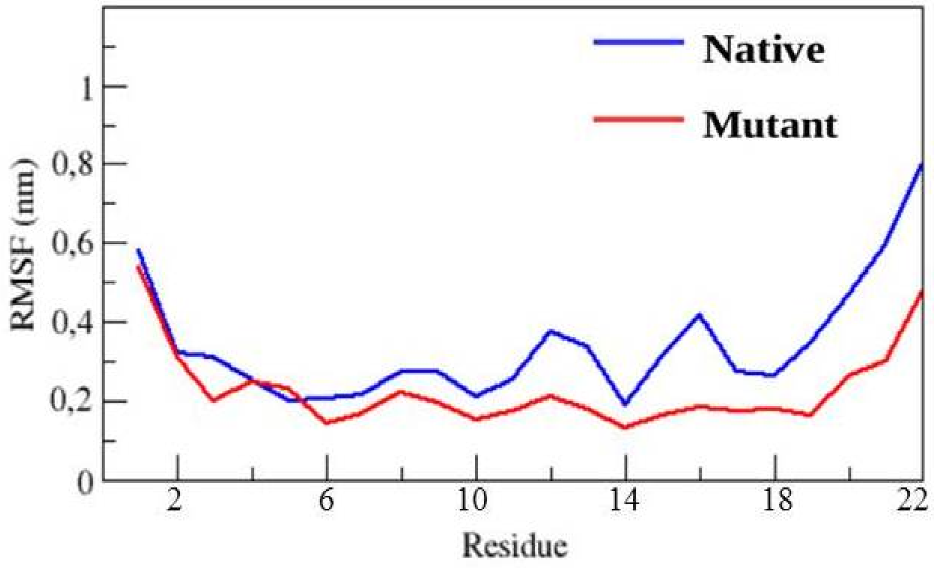

Figure S5.RMSF of Cα-atoms of native and mutant (F335Y) TM6 of P-glycoprotein at 300K.

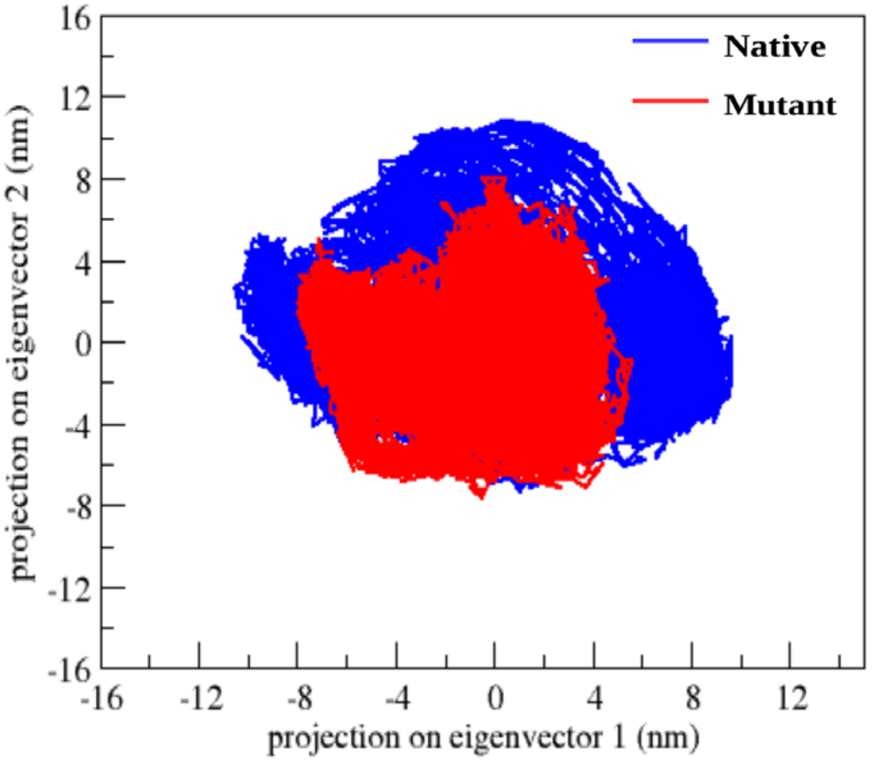

Figure S6.Projected motion of TM6 of P-glycoprotein in phase space along the first two principal eigenvectors at 300K.

References

1 Elstner, M., Frauenheim, T. & Suhai, S. An approximate DFT method for QM/MM simulations of biological structures and processes. *Journal of Molecular Structure: THEOCHEM* **632**, 29-41 (2003).

2 Zhang, Y. I-TASSER server for protein 3D structure prediction. *BMC bioinformatics* **9**, 40 (2008).

3 Laskowski, R. A., MacArthur, M. W., Moss, D. S. & Thornton, J. M. PROCHECK: a program to check the stereochemical quality of protein structures. *Journal of applied crystallography* **26**, 283-291 (1993).

4 Wiederstein, M. & Sippl, M. J. ProSA-web: interactive web service for the recognition of errors in three-dimensional structures of proteins. *Nucleic acids research* **35**, W407-W410 (2007).

5 Hess, B., Kutzner, C., Van Der Spoel, D. & Lindahl, E. GROMACS 4: algorithms for highly efficient, load-balanced, and scalable molecular simulation. *Journal of chemical theory and computation* **4**, 435-447 (2008).

6 MacKerell, A. D., Banavali, N. & Foloppe, N. Development and current status of the CHARMM force field for nucleic acids. *Biopolymers* **56**, 257-265 (2000).

7 Berendsen, H. J., Postma, J. P. M., van Gunsteren, W. F., DiNola, A. & Haak, J. Molecular dynamics with coupling to an external bath. *The Journal of chemical physics* **81**, 3684-3690 (1984).

8 Cheatham, T. I., Miller, J., Fox, T., Darden, T. & Kollman, P. Molecular dynamics simulations on solvated biomolecular systems: the particle mesh Ewald method leads to stable trajectories of DNA, RNA, and proteins. *Journal of the American Chemical Society* **117**, 4193-4194 (1995).

9 Amadei, A., Linssen, A. & Berendsen, H. Essential dynamics of proteins. *Proteins Structure Function and Bioinformatics*, 412-425 (1994).
